# Comparison of Observation Alone Versus Interventional Procedures in Hemodynamically Stable Patients With Pneumothorax: A Systematic Review and Meta-Analysis

**DOI:** 10.7759/cureus.58385

**Published:** 2024-04-16

**Authors:** Hatem Al Wahaibi, Ahmed Al Salmi, Abdullah Al Reesi, Mohamed Al Shamsi

**Affiliations:** 1 Emergency Medicine, Ibra Hospital, Ibra, OMN; 2 Emergency Medicine, Royal Hospital, Muscat, OMN; 3 Emergency Medicine, Windsor Regional Hospital, Ontario, CAN; 4 Emergency Medicine, Armed Forces Hospital, Muscat, OMN

**Keywords:** outcome analysis, primary pneumothorax, intercostal drainage, chest tube, pneumothorax ptx

## Abstract

Several studies indicate that observation alone is sufficient for the management of stable pneumothorax. To compare clinical efficacy, tolerability, and safety outcomes for treating hemodynamically stable adult patients with pneumothorax, the present review compared observation alone versus interventional procedures. We searched PubMed and Google Scholar from inception until June 24, 2020, for randomized controlled trials (RCTs) comparing observational therapy with conventional therapy for the treatment of adult pneumothorax. The pediatric age group and patients with tension pneumothorax were not included. Four hundred and forty-six patients were enrolled in three RCTs. The failure rate (relative risk (RR) 4.30; 95% CI = 0.23-81.82, p = 0.33) and mortality (RR 1.01; 95% CI = 0.31-3.33, p = 0.98) of observation were comparable to those of the chest tube. Chest tube and observation both carried comparable risks of complications, including tension pneumothorax and empyema (RR 3.15; 95% CI = 0.67-1) and (RR 1.55; 95% CI = 0.21-11.56, p = 0.67), respectively. Between chest tubes and observation, there was no statistically significant difference in the duration of hospital stay. We conclude that observation is as safe and effective at treating adult patients with stable pneumothorax as a chest tube.

## Introduction and background

A pneumothorax is an abnormal collection of air in the pleural space between the lung and the chest wall. The pneumothorax can be classified according to its cause and size. Spontaneous pneumothoraxes, which occur without thoracic trauma, are classified as primary or secondary [1]. Primary pneumothoraxes occur without clinically apparent lung disease, while secondary pneumothoraxes occur in patients with underlying lung disease, which most often is chronic obstructive pulmonary disease (COPD).

The American College of Chest Physicians (ACCP, 2001) and the British Thoracic Society (BTS, 2010) guidelines each have different techniques to determine the size of the pneumothorax [2, 3]. The ACCP classifies the size according to the distance from the lung apex to the ipsilateral thoracic cupola at the parietal surface, as determined by an upright standard radiograph. If the size of the pneumothorax is less than 3 cm, it is classified as small, and a large pneumothorax is more than or equal to 3 cm. The BTS classifies pneumothorax according to the distance from the chest wall to the outer edge of the lung at the level of the hilum. If the size of the pneumothorax is less than 2 cm, it is classified as small, and a large pneumothorax is more than or equal to 2 cm.

The incidence of primary spontaneous pneumothorax (PSP) admissions is 14.1 per 100,000 people age 15 years and older, with a higher rate for males (20.8) than females (7.6), reflecting an increase over earlier years [4]. The treatment of pneumothorax differs according to hemodynamic stability and size. According to BTS guidelines, any unstable, bilateral, or symptomatic pneumothorax should undergo active intervention, regardless of its size. Observation with supplemental oxygen is appropriate for small, stable pneumothoraxes. The observation may be a choice for selected asymptomatic patients with a large PSP, and it does not clarify this selection. Previous studies reported that conservative management of a larger pneumothorax is possible if there is no underlying lung disease [5]. A recently published study shows the safety of observation as a line of treatment for traumatic pneumothorax [6].

The clinical evidence to support observation as a safe and effective management method for pneumothorax needs more clarification. There was a previously published systematic review and meta-analysis comparing observation with conventional therapy, but it was not completed due to the lack of randomized controlled trials (RCTs) studies [7]. We conducted a systematic review and meta-analysis comparing observation with conventional therapy in the treatment of pneumothorax.

This article was previously submitted as a meeting abstract at the 2021 Bahrain Emergency Medical Conference (BEMC) on November 24, 2021.

## Review

Methods

The protocol for this systematic review followed the Preferred Reporting Items for Systematic Reviews and Meta-Analyses (PRISMA) guidelines. We last updated our search in March 2020 to ensure that there were no new trials that would meet the inclusion criteria of our systematic review and meta-analysis.

Systematic search

We searched PubMed and Google Scholar databases from inception until June 24, 2020, looking for RCTs that compared observational therapy with conventional therapy for the treatment of adult pneumothorax. Only studies published in English were included in our search. With the help of a medical librarian, we created the search strategy and added the following search terms: thoracostomy, chest tube thoracostomy, thoracotomy, pneumothorax, observation, and thoracentesis. To find synonyms, we looked through the Medical Subject Headings (MeSH). We looked for more pertinent studies in the full-text articles' references list.

The inclusion criteria based on population, intervention, control, and outcomes (PICO) criteria were as follows: P: stable patients with pneumothorax; I: use of a chest tube; C: closed monitoring following pneumothorax; O: chest tube insertion to treat desaturation and/or hemodynamic instability

Study selection

We included RCTs that randomized patients to observation and intervention, disregarding types of pneumothoraxes. We included studies of adult patients aged more than 18 years old. The primary investigator (HAW) screened all articles for eligibility. Then, two co-investigators (AAS and MAS) reviewed these articles for eligibility.

Data extraction and quality assessment

The data were extracted manually from articles into a Microsoft Excel (Microsoft Corp., Redmond, WA) datasheet. We extracted the following information from the included studies: title, publication year, sample size, objective, pneumothorax size, success, complications, mortality, and duration of hospitalization. We did not contact the study authors for clarification because all the data about the objectives are available in the papers.

The primary outcome was the failure rate in the observation and intervention groups. If the failure rate was not mentioned in the study, we extracted the success number from the total number of particular studies. The secondary outcomes were complications, mortality, and length of hospitalization. We limited the complication to tension pneumothorax and empyema as serious complications. We considered the mean of hospitalization days as a tool of comparison between the two groups.

The methodological quality of the included studies was independently evaluated by two authors (HAW and AAS) as recommended by the Cochrane Intervention System Evaluation Manual (Cochrane Handbook for Systematic Reviews of Interventions) [8]. If the two researchers disagreed about the methodological assessment, another researcher (MAS) was involved in concluding. The methodological assessment comprised of random sequence generation (selective bias), allocation concealment (selective bias), blinding of participants and personnel (performance bias), blinding outcome assessment (detection bias), incomplete outcome data (attrition bias), selective reporting (reporting bias), and other biases. A traffic light and a summary plot were plotted using the Cochrane risk-of-bias tool for randomized trials (RoB 2) tool [9].

Statistical analysis

We used RevMan 5.4 (The Cochrane Collaboration, Oxford, UK) software for meta-analysis. The data were manually inserted into the software [10]. Using the pooled baseline prevalence from the included trials' control arm, we computed the absolute effects. Using visual inspection of the forest plots, the Chi-squared test for homogeneity (where p<0.1 indicates significant heterogeneity), and the I2 statistic (where a value of 50% or higher was considered suggestive of potentially significant heterogeneity), we evaluated the degree of heterogeneity between trials.

Results

**Figure 1:**
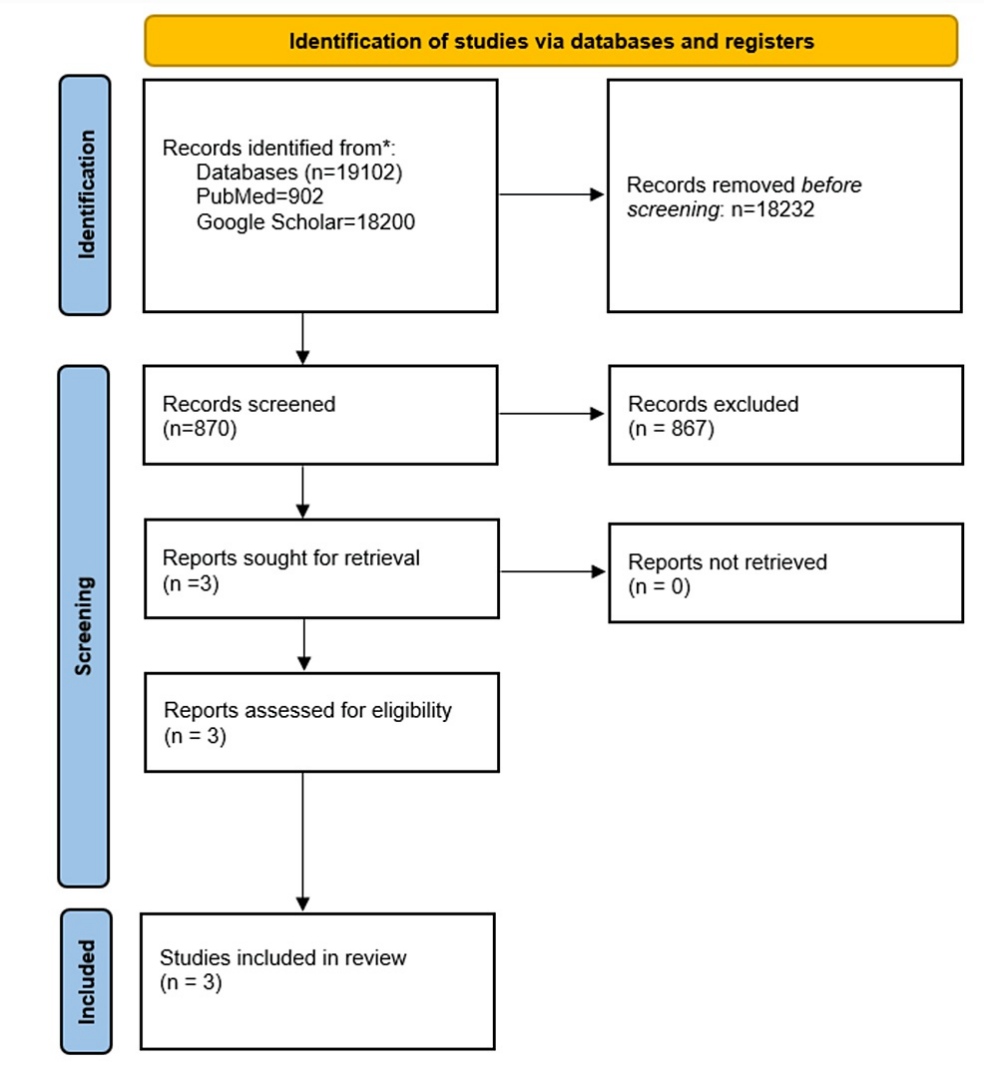
The PRISMA flowchart outlining the study selection process PRISMA: Preferred Reporting Items for Systematic Reviews and Meta-Analyses

From the 19,102 citations identified in the search (Figure 1), we excluded 870 duplicates and 18,229 citations after title and abstract screening. We assessed three full-text RCTs, and all of them are included in the review. There were 446 patients included in total. The study by Brown et al. [11], had 316 patients who had spontaneous pneumothorax detected by chest radiograph. In the study by Kirkpatrick et al. [12], there were 90 patients, and a total of 130 patients had a traumatic occult pneumothorax. In the study by Enderson et al. [13], there were 40 patients. 

The baseline characteristics of the included trials are summarized in Table 1. The numbers of patients were almost equal between observation and chest tubes. The type of intervention was tube thoracostomy in all studies.

**Table 1:** Summary of the included studies pntx: pneumothorax

Author/year	Sample size (n)	Observation						Intervention						
		No. of patients	Need for intervention	Tension Pntx	Empyema	Mortality	Hospitalization days	No. of patients	Type of intervention	Further intervention	Tension pntx	Empyema	Mortality	Hospitalization days
Brown et al., 2020 [11]	316	162	25	2	1	1	0.2	154	Tube thoracostomy	No	1	1	0	3.8
Kirkpatrick et al., 2012 [12]	90	50	10	1	0	4	18	40	Tube thoracostomy	No	0	0	4	16
Enderson et al., 1993 [13]	40	21	8	3	1	0	17.6	19	Tube thoracostomy	No	0	0	0	12.9

For PSP, Brown et al. [11] evaluated the observation approach in comparison to interventional treatment. It was a multicenter, open-label, non-inferiority trial. Patients with first-known, unilateral, moderate-to-large primary spontaneous pneumothorax between the ages of 14 and 50 were recruited. A total of 316 patients were identified, of whom 154 were assigned to the intervention group and 162 to the conservation group at random. The main result of their 12-month follow-up on those patients was lung re-expansion in just eight weeks. Based on chest radiography, the Collins method indicated that this pneumothorax was 32% or higher (sum of interpleural distances, >6 cm). Pneumothorax expansion or clinical deterioration was monitored in the conservative group. A small-bore (≤12 French) chest tube using the Seldinger technique was inserted and secured to an underwater seal for all patients in the intervention group. The tube was removed after four hours if the patient showed no symptoms and the pneumothorax had resolved. In this study, 39 out of 154 patients in the interventional group underwent additional intervention, and 25 out of 162 patients (15.4%) in the observational group underwent interventions to manage the pneumothorax. As a result, Brown et al. concluded that the observation has a lower risk of serious adverse events and is equally effective in managing spontaneous pneumothorax as the chest tube.

The study by Kirkpatrick et al. [12] included occult pneumothorax, defined as a pneumothorax on computed tomography (CT), which was not suspected on preceding supine anteroposterior chest radiographs. The study was conducted in 24 trauma centers in Calgary and Quebec over 20 months. It included any size of occult traumatic pneumothorax for randomization, and it included patients aged 18 years old or older. They found 90 patients with traumatic occult pneumothorax requiring positive pressure ventilation (PPV). Those patients were randomized to tube thoracostomy (40 patients) and observation (90 patients). Other methods of drainage, like percutaneous catheters, were counted in the tube thoracostomy group. The primary outcome was a composite of respiratory distress (RD) (need for urgent pleural drainage, acute/sustained increases in oxygen requirements, ventilator desynchrony, and/or charted respiratory events). This study compared the observation with a chest tube in the management of patients with a traumatic occult pneumothorax who underwent PPV in an ICU, and this study showed the failure of observation; hence, 20% of observed patients ended with drainage for different reasons, but there was no increase in mortality, ventilation needs, or hospital stay. Although this study showed that observation can be a choice of pneumothorax management, there was still a risk of bias against observation; hence, all patients received PPV, which can increase complications, including tension pneumothorax.

The study by Enderson et al. [13] was conducted at the University of Tennessee Medical Center over 18 months. It included any patient with a normal chest radiograph who was found to have pneumothorax in abdominal CT. The abdominal computed CT was conducted for different traumatic indications, and this CT was able to detect inferior occult pneumothoraxes. They found 40 eligible patients, and those 40 patients were distributed randomly to observation and tube thoracostomy. The tube thoracostomy was a 36F tube placed through the fifth intercostal space in the midaxillary line. The size of the pneumothorax was not mentioned in detail. They did not insert a thoracostomy tube if anyone from the observation group required PPV unless they developed complications or progression of pneumothorax. The main objective was to test the hypothesis that tube thoracostomy is unnecessary for occult pneumothoraxes, regardless of whether the patient required PPV or not. In this study, eight out of 21 needed further intervention by insertion of a chest tube, but there are no data mentioned about the intervention group, so we considered it zero.

Risk of bias assessment

**Figure 2:**
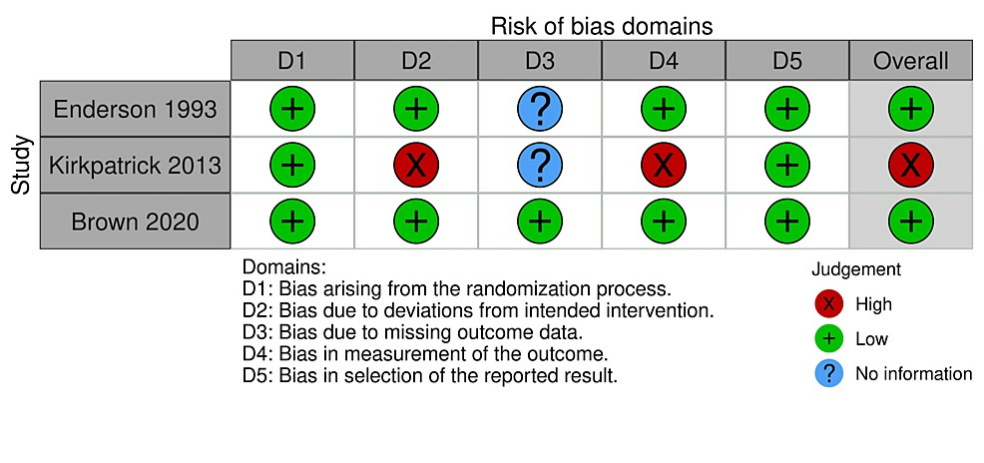
Traffic light plot References [13,12,11]

**Figure 3:**
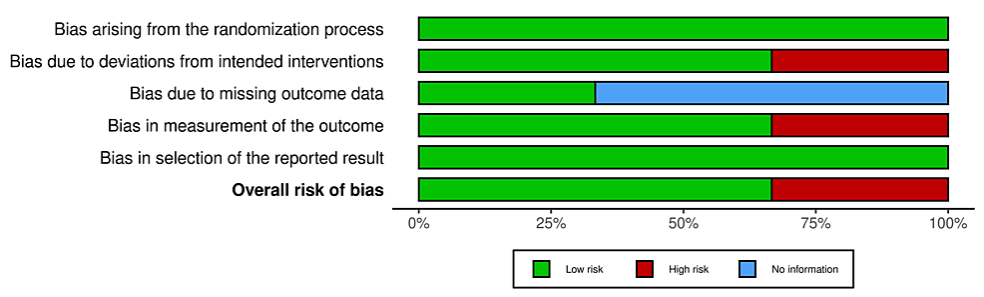
Summary plot

The traffic light plot in Figure 2 and the summary plot in Figure 3 illustrate the risk of bias within the trials based on ROB2. In all three trials, the randomization process's bias was minimal [11-13]. Two studies [11, 13] found low bias resulting from deviations from intended interventions (allocation concealment), while one study [12] found high bias. One study [12] found low bias resulting from missing outcome data, while two other studies [12, 13] found no information. Two studies [11, 13] found bias in outcome measurement to be low, while one study [9] found high outcome measurement bias. In all three studies, bias resulting from the results' selection and reporting was low [11-13]. Overall bias was low in two studies [11, 13] and high in one study [12].

Efficacy outcomes

Rate of Failure Between Observation and Intervention

**Figure 4:**
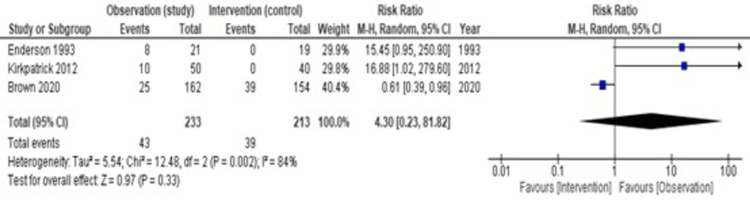
A forest plot showing the rate of failure between observation and intervention [13,12,11]

Forty-three patients out of 233 in the observation group required further intervention. On the other hand, 39 patients who had spontaneous pneumothorax required further surgical intervention in addition to a chest tube, and traumatic pneumothorax didn’t require any intervention. Figure 4 shows heterogeneity (I2) is 84%; hence, we have used a random effect model. The pooled estimate of the risk ratio is 4.30 (95% CI = 0.23-81.82, p = 0.33). Therefore, we can conclude that there is no statistically significant difference in the risk of failure between the intervention group and the observation group.

Complication Rates: Tension Pneumothorax and Empyema

**Figure 5:**
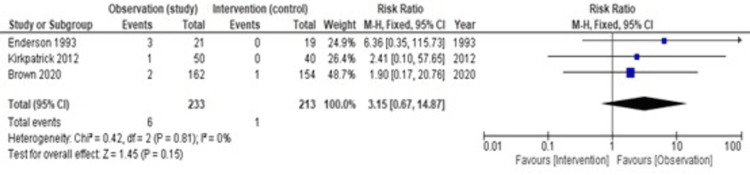
A forest plot showing complication rates: tension pneumothorax [13,12,11]

We chose tension pneumothorax and empyema as complications exclusively because tension pneumothorax is the most serious complication in both groups, and empyema is a serious complication of unnecessary introduction to the sterile pleural cavity. Tension pneumothoraxes were seen more in the observation group compared with the intervention group. Figure 5 shows heterogeneity (I2) is 0%, hence we have used a fixed effect model. The pooled estimate of the risk ratio is 3.15 (95% CI = 0.67-14.87, p = 0.15). Therefore, we can conclude that there is no statistically significant difference in the risk of tension pneumothorax between the intervention group and the observation group.

**Figure 6:**
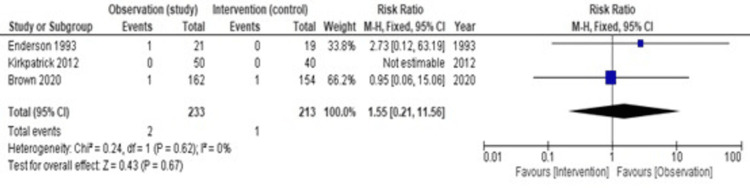
A forest plot showing complication rates: empyema [13,12,11]

Empyema occurred more in the observation group but was not statistically significant. Figure 6 shows heterogeneity (I2) is 0%, hence we have used a fixed effect model. The pooled estimate of the risk ratio is 1.55 (95% CI = 0.21-11.56, p = 0.67).

Mortality

**Figure 7:**
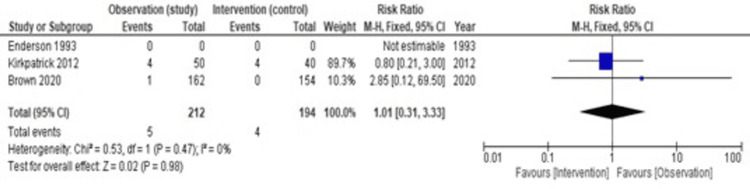
A forest plot comparing mortality between two groups [13,12,11]

There were no data about mortality in the Enderson study [13]. Therefore, we included the Brown and Kirkpatrick studies to assess the mortality rate between the observation and intervention groups, as shown in Figure 7. In the Brown study, the death in the observation group was suicidal and happened after the complete resolution of pneumothorax. Although we counted this death, we can conclude that there is no statistically significant difference in the risk of mortality between the intervention group and the observation group. Heterogeneity (I2) is 0%; hence, we have used a fixed effect model. The pooled estimate of the risk ratio is 1.01 (95% CI = 0.31-3.33, p = 0.98).

Hospitalization Length

**Figure 8:**
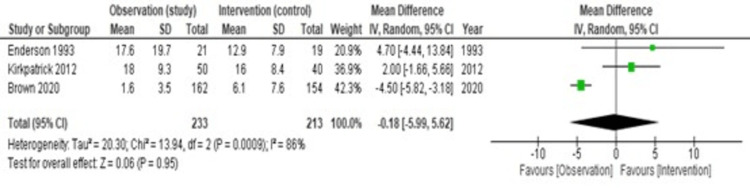
A forest plot comparing the hospitalization length between the two groups References [13,12,11]

The period of hospitalization was shorter in the observational group in the study by Brown et al. [11], but both other studies show longer hospitalization in the observational group. Figure 8 shows heterogeneity (I2) is 86%, which exceeds the limit of 50%, and hence we have used a random effect model. The pooled estimate of the mean difference is -0.18 (95% CI = -5.99-5.62, p = 0.95). Therefore, we can conclude that there is no statistically significant difference in the mean number of hospital days between the intervention group and the observation group.

Discussion

This systematic review and meta-analysis demonstrates the safety of a conservative strategy as a choice of treatment for different types of pneumothoraxes. Although the pooled estimate of mortality is in the middle of the forest plot, we believe that the risk is less than this because the mortality in the study carried out by Brown et al. [11] was suicidal and unrelated to pneumothorax treatment. This suicidal death in the observation group of the Brown et al. study [11] affected the pooled estimation.

The available literature suggests the effectiveness of observation as the treatment of pneumothorax in either traumatic or spontaneous circumstances [14-18]. The worry about observation with several clinicians is that observation has a failure rate higher than tube thoracostomy [19-23]. Our study shows no statistical difference between observation and tube thoracotomy in the rate of failure. This finding supports the use of observation for pneumothorax management.

Tension pneumothorax is a serious complication of pneumothorax that should be considered when managing pneumothorax, especially while considering PPV [24-27]. Observation carries the risk of expanding the pneumothorax to tension the pneumothorax. However, there was no evidence to support the observation of increased tension pneumothorax incidence compared to tube thoracostomy. This study shows there is no statistical difference between the two groups in the incidence of tension pneumothorax. Although empyema occurred more in intervention because of the introduction of microbes by tube thoracostomy to the pleural cavity, this study shows empyema occurs more in observation compared to intervention, but it is not significantly different.

This study shows hospitalization length is shortened with time in the observational group. Enderson et al. [12] and Kirkpatrick et al. [13] demonstrated that hospitalization length was longer with tube thoracostomy, but Brown et al. [11] demonstrated different results because of numerous subsequent studies on the safety of observation to manage pneumothorax. Overall, we found no significant difference in hospitalization length between observation and tube thoracostomy.

This meta-analysis has some limitations. Firstly, we only found three RCTs comparing observation with conventional tube thoracotomy against what we expected. Secondly, two RCTs [12, 13] included occult pneumothorax, which has less risk of complications and failure compared to large pneumothorax, but most of the patients in both studies underwent PPV. Positive pressure ventilation increased the risk of complications and treatment failure in pneumothorax.

## Conclusions

We found no significant difference between observation and tube thoracostomy in the treatment of an unspecified pneumothorax. However, this conclusion was derived from three studies, which are quite small in number. Furthermore, the failure rate of the treatment, risk of tension pneumothorax and empyema, mortality, and hospitalization length are comparable between observation and tube thoracostomy. Given the paucity of RCTs that compare observation with tube thoracotomy, additional RCTs are needed to solidify the observation strategy as the first line of management for stable pneumothorax.
